# The Histone H3K79 Methyltransferase Dot1L Is Essential for Mammalian Development and Heterochromatin Structure

**DOI:** 10.1371/journal.pgen.1000190

**Published:** 2008-09-12

**Authors:** Brendan Jones, Hui Su, Audesh Bhat, Hong Lei, Jeffrey Bajko, Sarah Hevi, Gretchen A. Baltus, Shilpa Kadam, Huili Zhai, Reginald Valdez, Susana Gonzalo, Yi Zhang, En Li, Taiping Chen

**Affiliations:** 1 Epigenetics Program, Novartis Institutes for Biomedical Research, Cambridge, Massachusetts, United States of America; 2 Department of Radiation Oncology, Radiation and Cancer Biology Division, Washington University School of Medicine, St. Louis, Missouri, United States of America; 3 Developmental and Molecular Pathways, Novartis Institutes for Biomedical Research, Cambridge, Massachusetts, United States of America; 4 Analytical Sciences, Novartis Institutes for Biomedical Research, Cambridge, Massachusetts, United States of America; 5 Howard Hughes Medical Institute, Chevy Chase, Maryland, United States of America; 6 Department of Biochemistry and Biophysics, Lineberger Comprehensive Cancer Center, University of North Carolina at Chapel Hill, Chapel Hill, North Carolina, United States of America; Medical Research Council Human Genetics Unit, United Kingdom

## Abstract

Dot1 is an evolutionarily conserved histone methyltransferase specific for lysine 79 of histone H3 (H3K79). In *Saccharomyces cerevisiae*, Dot1-mediated H3K79 methylation is associated with telomere silencing, meiotic checkpoint control, and DNA damage response. The biological function of H3K79 methylation in mammals, however, remains poorly understood. Using gene targeting, we generated mice deficient for *Dot1L*, the murine *Dot1* homologue. *Dot1L*-deficient embryos show multiple developmental abnormalities, including growth impairment, angiogenesis defects in the yolk sac, and cardiac dilation, and die between 9.5 and 10.5 days post coitum. To gain insights into the cellular function of Dot1L, we derived embryonic stem (ES) cells from *Dot1L* mutant blastocysts. *Dot1L*-deficient ES cells show global loss of H3K79 methylation as well as reduced levels of heterochromatic marks (H3K9 di-methylation and H4K20 tri-methylation) at centromeres and telomeres. These changes are accompanied by aneuploidy, telomere elongation, and proliferation defects. Taken together, these results indicate that Dot1L and H3K79 methylation play important roles in heterochromatin formation and in embryonic development.

## Author Summary

Histone methylation plays a critical role in the regulation of gene expression and chromatin structure. Among the sites of histone methylation, lysine 79 of histone H3 (H3K79) is unique in that it is not located within the H3 N-terminal tail but in the globular domain. Our knowledge about H3K79 methylation comes primarily from studies in yeast. This study focuses on the role of H3K79 methylation in mammalian development and cellular function. We show that genetic disruption of *Dot1L*, the only known H3K79 methyltransferase gene in mouse, results in embryonic lethality. At the cellular level, *Dot1L* deficiency leads to alterations in constitutive heterochromatin, accompanied by telomere elongation, aneuploidy, and proliferation defects. Our work represents a key step toward understanding the function of H3K79 methylation in mammals.

## Introduction

Histones are subject to a variety of post-translational modifications, including acetylation, phosphorylation, ubiquitination, and methylation. These modifications dictate chromatin structure by affecting the recruitment of nonhistone proteins and/or the interactions between nucleosomes [1],[2]. Heterochromatin is associated with high levels of methylation at H3K9, H3K27, and H4K20 and low levels of acetylation, whereas actively transcribed euchromatin is typically enriched with acetylation and methylated H3K4, H3K36, and H3K79.

Most histone H3 modifications occur on residues within the N-terminal tail. In contrast, H3K79 is located in a loop within the globular domain, exposed on the nucleosome surface. The yeast Dot1 and its homologues in other species are the only known H3K79 methyltransferases [3]–[5]. Unlike other histone lysine methyltransferases, Dot1 family members do not have a SET domain [3]–[5]. Instead, their catalytic domain contains conserved sequence motifs characteristic of class I methyltransferases such as DNA methyltransferases (DNMTs) and the protein arginine methyltransferase PRMT1 [6].

Dot1 was initially identified as a disruptor of telomeric silencing in *Saccharomyces cerevisiae* [3]. Subsequent studies showed that both overexpression and inactivation of Dot1 as well as mutations at H3K79 all lead to loss of telomeric silencing [7]–[9]. Although the mechanisms by which Dot1 affects telomere structure and function are not fully understood, it is believed that H3K79 methylation plays an important role in restricting the Sir proteins at heterochromatic regions [7],[8],[10]. Dot1-dependent H3K79 methylation has also been shown to be involved in meiotic checkpoint control and in G1 and S phase DNA damage checkpoint functions of Rad9 in yeast [11],[12].

H3K79 methylation is also a widespread histone modification in mammalian cells [4]. Abnormal H3K79 methylation has been linked to leukemogenesis in humans [13],[14]. However, the biological function of H3K79 methylation in mammals remains largely unknown. Here we generated a mouse line containing a null mutation in *Dot1L*, the murine *Dot1* homologue, and investigated the role of *Dot1L* and H3K79 methylation in embryonic development and cellular function. We provide evidence that *Dot1L* is required for embryogenesis and for the integrity of constitutive heterochromatin at the cellular level.

## Results

### Generation of *Dot1L* Conditional and Null Alleles in Mice

**Figure 1:**
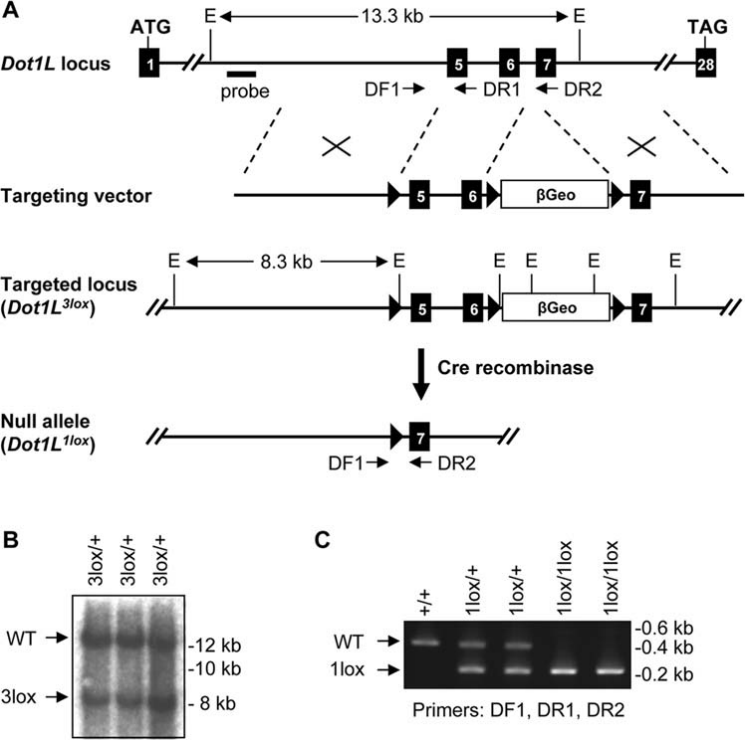
Generation of mutant *Dot1L* alleles in mice. (A) Schematic depiction of the strategy used to generate the *Dot1L^3lox^* and *Dot1L^1lox^* alleles. The exons are numbered. The locations of the Southern probe and PCR primers (DF1, DR1, and DR2) used for genotyping, as well as the sizes of the diagnostic fragments recognized by the Southern probe, are indicated (E, *Eco*RI). *loxP* sites are shown as triangles. (B) Southern blot analysis of *Eco*RI-digested genomic DNA probed with an 860-bp 5′ probe external to the targeting vector. The presence of the 8.3-kb band confirms homologous recombination. (C) PCR genotyping of DNA from ES cells. WT, 485 bp; 1lox, 233 bp.

To target the *Dot1L* gene, we constructed a targeting vector in which a 2.3-kb genomic region containing exons 5 and 6 and a promoterless β-geo selection cassette were flanked, respectively, by three *loxP* sites (Figure 1A). Exons 5 and 6 encode 108 amino acids that form several conserved motifs in the Dot1L catalytic domain, including the SAM-binding motif and motifs X, I, and II [6]. Since mutations of conserved residues within motif I abolish the methyltransferase activity of Dot1L [4], we predicted that deletion of exons 5 and 6 would inactivate *Dot1L*.

ES cells were transfected with the targeting vector and selected in G418-containing medium. Clones with homologous recombination were identified by Southern blot analysis with a 5′ external probe (Figure 1B). Three of these clones, referred to as *Dot1L^3lox/+^*, were injected into blastocysts to generate chimeric mice, which subsequently transmitted the mutant allele to their offspring. Deletion of the β-geo cassette plus exons 5 and 6 was achieved by breeding the *Dot1L^3lox^* allele into mice expressing Cre recombinase in the germ line. The resulting null allele is referred to as *Dot1L^1lox^* (Figure 1A). Genotypes were determined using PCR (Figure 1C).

### *Dot1L* Is Essential for Embryonic Development

**Figure 2:**
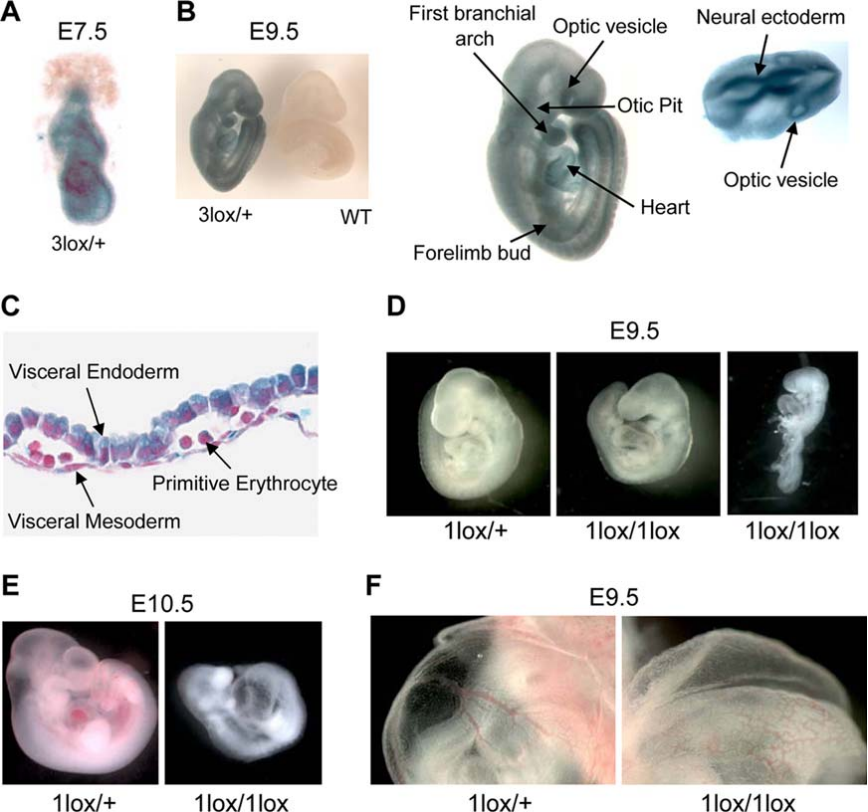
Essential role for Dot1L in mouse embryonic development. (A) A representative X-gal stained 7.5-dpc *Dot1L^3lox/+^* embryo demonstrating ubiquitous *Dot1L* transcription throughout the embryo. (B) A representative X-gal stained 9.5-dpc *Dot1L^3lox/+^* embryo demonstrating ubiquitous *Dot1L* transcription throughout the embryo with elevated *Dot1L* expression in the indicated regions. (C) A representative X-gal stained 9.5-dpc *Dot1L^3lox/+^* yolk sac demonstrating *Dot1L* transcription in visceral endoderm, visceral mesoderm, and primitive erythrocytes. (D) Representative pictures of 9.5-dpc *Dot1L^1lox/+^* and *Dot1L^1lox/1lox^* embryos. *Dot1L^1lox/+^* embryos (left) were indistinguishable from wild-type embryos. Most *Dot1L^1lox/1lox^* embryos were undersized, had an enlarged heart (cardiac dilation) and stunted tail (center), while approximately 15% exhibited developmental arrest at E8.5 (right). (E) Representative pictures of a 10.5-dpc *Dot1L^1lox/1lox^* embryo (right) and a heterozygous littermate (left). (F) Representative pictures showing the yolk sac vasculature of 9.5-dpc *Dot1L^1lox/+^* (left) and *Dot1L^1lox/1lox^* (right) embryos. The vasculature of the *Dot1L^1lox/1lox^* yolk sac is thinner and less organized than that of the heterozygous littermate.

We first determined the expression of *Dot1L* during embryonic development, taking advantage of the fact that cells containing the *Dot1L^3lox^* allele express *lacZ* under the control of the endogenous *Dot1L* promoter. We conducted X-gal staining on *Dot1L^3lox/+^* heterozygous embryos and wild-type littermates at different stages of development. *Dot1L* expression is ubiquitous as early as 7.5-dpc (the earliest time point tested, Figure 2A). At 9.5-dpc, *Dot1L* expression remains ubiquitous and areas of elevated expression are apparent. Tissues that demonstrate high levels of *lacZ* staining include the optic vesicle, the first branchial arch, the limb buds, the heart, the otic pit, and the neural ectoderm (Figure 2B). *Dot1L* is also expressed at high levels in extra-embryonic tissues, including the visceral endoderm and visceral mesoderm of the yolk sac, and in primitive erythrocytes (Figure 2C). Similar *lacZ* staining patterns are observed in embryos harvested at 10.5-dpc, 11.5-dpc, and 12.5-dpc (data not shown), suggesting that *Dot1L* is broadly expressed during embryonic development.

*Dot1L^1lox/+^* mice were grossly normal and fertile. However, intercrosses of *Dot1L^1lox/+^* mice produced no viable *Dot1L^1lox/1lox^* homozygous offspring, suggesting embryonic lethality (Table 1). *Dot1L^1lox/1lox^* embryos harvested at 8.5-dpc were indistinguishable from wild-type and *Dot1L^1lox/+^* littermates (data not shown). At 9.5-dpc, *Dot1L^1lox/1lox^* embryos were smaller than littermates, had enlarged hearts and stunted tails on gross observation when viewed under a dissecting microscope (Figure 2D, center). Approximately 15% of the *Dot1L^1lox/1lox^* embryos demonstrated a severe phenotype, exhibiting developmental arrest at E8.5 (Figure 2D, right). Histological examination of 9.5-dpc *Dot1L^1lox/1lox^* embryo sections revealed focal areas of extensive apoptosis, but no obvious structural defects (Figure S1). At 10.5-dpc, the percentage of viable *Dot1L^1lox/1lox^* embryos was substantially below the expected Mendelian ratio (Table 1), suggesting that many of the *Dot1L^1lox/1lox^* embryos die during this time interval. The few that survived to this stage exhibited developmental arrest at E9.5 and severe cardiac dilation (Figure 2E). No *Dot1L^1lox/1lox^* embryos survived beyond 10.5-dpc (Table 1).

**Table 1:** Dot1L deficiency results in embryonic lethality.

	8.5-dpc	9.5-dpc	10.5-dpc	11.5-dpc	12.5-dpc	Birth
Litters	8	12	5	3	2	6
Embryos	67	105	37	28	16	58
*Dot1L^+/+^*	14 (21%)	26 (25%)	11 (30%)	8 (29%)	3 (19%)	20 (34%)
*Dot1L^1lox/+^*	34 (51%)	50 (48%)	17 (46%)	13 (46%)	5 (31%)	38 (66%)
*Dot1L^1lox/1lox^*	14 (21%)	20 (19%)	3 (8%)	0 (0%)	0 (0%)	0 (0%)
resorbed	5 (7%)	9 (8%)	6 (16%)	7 (25%)	8 (50%)	NA

The numbers and percentages of *Dot1L^+/+^*, *Dot1L^1lox/+^*, *Dot1L^1lox/1lox^*, and resorbed embryos harvested at the indicated time points are shown. NA: not applicable.

As stunted growth and enlarged heart are phenotypes that often occur as a result of defects in extraembryonic tissues, we examined the yolk sac and placenta of 9.5-dpc *Dot1L^1lox/1lox^* embryos. While the placenta showed no obvious defects, the yolk sac exhibited abnormal vascular morphology. The yolk sac vasculature was present and contained primitive erythrocytes, but was frequently underdeveloped and disorganized when compared to control littermates (Figure 2F). These observations indicate that, in the absence of Dot1L, vasculogenesis took place in the yolk sac but angiogenesis was defective.

### *Dot1L* Deficiency in ES Cells Results in Growth Defects

**Figure 3:**
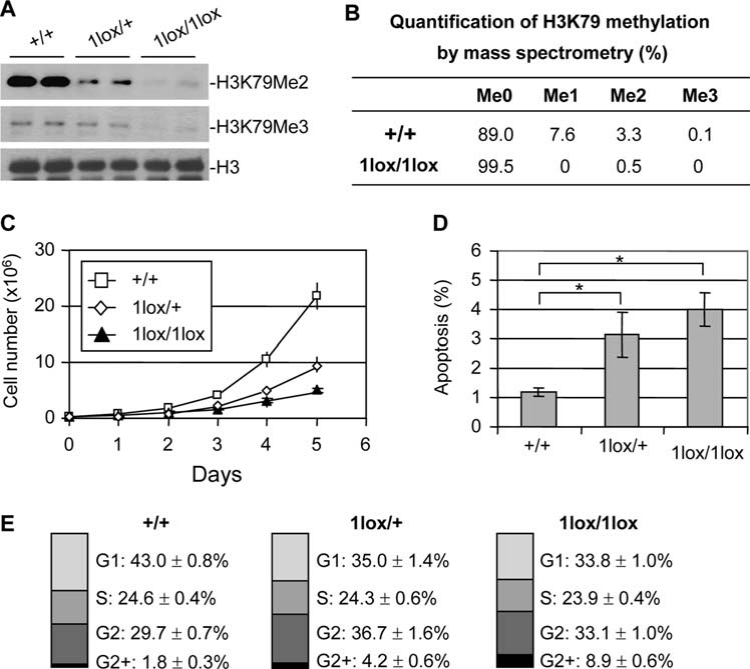
Phenotypic analysis of *Dot1L* mutant ES cells. (A) Western blot analysis using extracts from ES cell lines of the indicated *Dot1L* genotypes and antibodies specific for di-, and tri-methylated H3K79. Total histone H3 was used as a loading control. (B) Analysis of H3K79 methylation by mass spectrometry. Quantification of different forms of H3K79 methylation was obtained by comparing the extracted ion chromatogram (EIC) intensity of the ion signals corresponding to the unmodified (Me0), mono-methylated (Me1), di-methylated (Me2), and tri-methylated (Me3) K79-containing peptides. (C) The proliferation of *Dot1L^+/+^*, *Dot1L^1lox/+^* and *Dot1L^1lox/1lox^* ES cells was determined by doing cell counts every 24 hours for five days. Cells were grown in triplicate, and data shown is representative of three independent experiments. (D) The percentages of apoptotic cells in *Dot1L^+/+^*, *Dot1L^1lox/+^* and *Dot1L^1lox/1lox^* ES cell cultures. The asterisk indicates P<0.05 (Student t-test). ES cells were stained with propidium iodide (PI) and PE conjugated anti-annexin V antibodies and analyzed by FACS. Apoptotic cells were annexin V positive and PI negative. Cells were grown in triplicate, and data shown are representative of two independent experiments. (E) The percentages of each cell cycle stage in *Dot1L^+/+^*, *Dot1L^1lox/+^* and *Dot1L^1lox/1lox^* ES cell cultures as determined by PI staining and FACS.

To investigate the cellular function of Dot1L, we derived *Dot1L* mutant ES cells from blastocysts produced from intercrosses of *Dot1L^1lox/+^* mice. Two *Dot1L^1lox/1lox^* and multiple *Dot1L^1lox/+^* and *Dot1L^+/+^* lines were established. As expected, H3K79 di- and tri-methylation was greatly reduced in *Dot1L^1lox/1lox^* cells compared to *Dot1L^+/+^* cells (Figure 3A). *Dot1L^1lox/+^* cells had intermediate levels of H3K79 di- and tri-methylation, indicating haploinsufficiency of Dot1L (Figure 3A). Surprisingly, Western blot analysis using a “mono methyl H3K79” antibody (ab2886, Abcam) detected no change in signal intensity in *Dot1L* mutant ES cell lines (data not shown). To verify the results, we carried out mass spectrometry. In wild-type ES cells, ∼11% of histone H3 showed K79 methylation, among which mono-, di-, and tri-methylation accounted for ∼70%, ∼30%, and less than 1%, respectively. In *Dot1L^1lox/1lox^* ES cells, H3K79 mono- and tri-methylation was absent although trace amount of di-methylation was detected (Figure 3B and Figure S2). We therefore concluded that the Western blot result showing no alteration in H3K79 mono-methylation in the absence of Dot1L was an artifact due to nonspecific recognition of histone H3 by the “mono methyl H3K79” antibody. The low level of H3K79 di-methylation detected in *Dot1L^1lox/1lox^* samples could be from feeder cells present in the culture or due to incomplete inactivation of *Dot1L*. Taken together, these results indicated that Dot1L is most likely the sole H3K79 methyltransferase in mice.

*Dot1L* mutant ES cells maintained an undifferentiated state, as judged by morphology and ES cell markers such as Oct4 and Nanog (data not shown). We investigated whether Dot1L deficiency affects ES cell growth. We plated 3×10^5^ *Dot1L^+/+^*, *Dot1L^1lox/+^*, and *Dot1L^1lox/1lox^* ES cells in standard ES cell medium and monitored proliferation. By 24 hours, the number of *Dot1L^+/+^* ES cells (7.3×10^5^) was significantly higher than the number of *Dot1L^1lox/+^* or *Dot1L^1lox/1lox^* cells (4.2×10^5^ and 3.6×10^5^ respectively, P<0.05, Figure 3C). Over the 5 days examined, *Dot1L^+/+^*, *Dot1L^1lox/+^*, and *Dot1L^1lox/1lox^* ES cells had average doubling times of 16, 22, and 26 hours, respectively. The fact that both *Dot1L^1lox/+^* and *Dot1L^1lox/1lox^* cells exhibited growth defects showed the importance of *Dot1L* gene dosage in cellular function. The reduction of H3K79 methylation in *Dot1L^1lox/+^* cells (haploinsufficiency) suggested that Dot1L level is relatively low. Interestingly, *Dot1L^1lox/+^* mice were apparently normal despite the defects in *Dot1L^1lox/+^* ES cells. It is possible that 50% of Dot1L can barely maintain normal cellular function under ideal conditions (e.g. in vivo) but is not sufficient to do so under suboptimal conditions (e.g. in culture).

We next examined apoptosis and cell cycle status of the *Dot1L* mutant ES cells. Annexin V staining revealed that 4.0% of the *Dot1^1lox/1lox^* ES cells and 3.1% of the *Dot1L^1lox/+^* ES cells were annexin V positive, while only 1.2% of the *Dot1L^+/+^* ES cells were annexin V positive (Figure 3D). This indicates that more than twice as many of the *Dot1L* mutant ES cells were undergoing apoptosis compared to wild-type ES cells. Furthermore, cell cycle analysis by propidium iodide staining revealed an elevated percentage of G2 cells and a reduced percentage of G1 cells among the *Dot1L* mutant ES cells when compared to the wild-type ES cells (Figure 3E). These results suggest that both elevated apoptosis and G2 cell cycle arrest contribute to the reduced growth rate of *Dot1L* mutant ES cells.

### *Dot1L*-Deficient ES Cells Show Telomere Elongation and Aneuploidy

**Figure 4:**
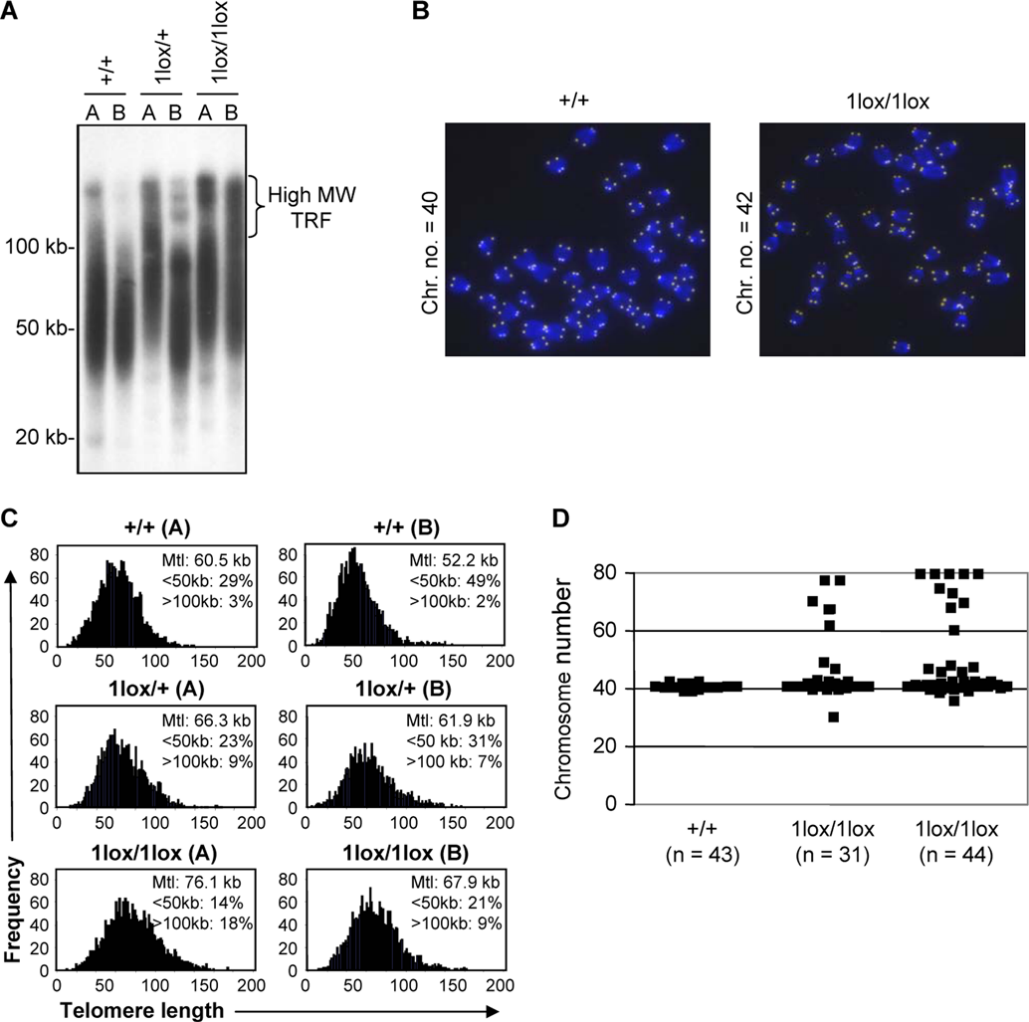
Telomere elongation and aneuploidy in *Dot1L*-deficient ES cells. (A) Telomere restriction fragment (TRF) analysis upon *Mbo*I digestion of genomic DNA from two independent ES cell clones of each of the genotypes: *Dot1L^+/+^*, *Dot1L^1lox/+^* and *Dot1L^1lox/1lox^*. Note the presence of high molecular weight TRFs in *Dot1L^1lox/1lox^* cells, which correspond to longer telomeres. (B) Representative images generated during the Q-FISH assay showing metaphase spreads from *Dot1L^+/+^* and *Dot1L^1lox/1lox^* ES cells labeled with a telomere-specific fluorescent probe. (C) Telomere length distribution of two independent ES cell clones of each of the *Dot1L* genotypes as determined by Q-FISH. Twenty metaphases of each ES cell clone were analyzed. Note the increase in mean telomere length (Mtl) in both clones of *Dot1^1lox/1lox^* cells and the intermediate phenotype of *Dot1^1lox/+^* lines. The percentages of telomeres below 50 kb and above 100 kb in length are indicated. (D) Scatter plot of the chromosome number of a *Dot1L^+/+^* ES cell line and two *Dot1L^1lox/1lox^* cell lines. Chromosome number was determined by manually counting chromosomes in chromosome spreads. Each point represents the chromosome number of a single cell (n represents the number of metaphase cells counted).

Dot1-deficient *S. cerevisiae* show telomere elongation and defects in telomere silencing [3]. We therefore evaluated the effect of Dot1L inactivation on telomere length. First, we used Southern blot terminal restriction fragment (TRF) analysis to estimate telomere length in two ES cell lines of each *Dot1L* genotype. Both *Dot1L^1lox/1lox^* lines and one of the *Dot1L^1lox/+^* lines showed telomere elongation, as evidenced by the presence of high molecular weight (MW) TRFs and the increase in the lengths of bulk TRFs compared to wild-type controls (Figure 4A). Next, we carried out quantitative fluorescence *in situ* hybridization (Q-FISH) using a telomere-specific probe to determine the mean telomere length (mtl) and the distribution of telomere lengths for each cell line (Figure 4, B and C). Consistent with the TRF results, both *Dot1L^1lox/1lox^* lines had higher mtl, greater percentages of elongated (>100 kb) telomeres, and reduced percentages of short (<50 kb) telomeres compared to *Dot1L^+/+^* lines (Figure 4C). The heterozygous ES cells again showed an intermediate phenotype (Figure 4C).

Examination of the Q-FISH samples revealed frequent aneuploidy in *Dot1L*-deficient cells (Figure 4B). To further investigate this phenotype, we prepared metaphase chromosome spreads from *Dot1L^1lox/1lox^* and *Dot1L^+/+^* ES cells and examined them for chromosomal defects. *Dot1L^+/+^* cells were karyotypically stable, as the vast majority had 40 chromosomes. In contrast, over 40% of metaphase *Dot1L^1lox/1lox^* cells were aneuploid. Most of the aneuploid cells showed gain of chromosomes and some ended up being tetraploid (Figure 4, B and D). Aside from aneuploidy, no obvious chromosomal abnormalities were frequently observed in *Dot1L*-deficient cells (Figure 4B). These results point to defects in chromosome segregation in the absence of Dot1L.

### Aberrant Telomere Elongation in *Dot1L*-Deficient Cells Correlates with Activation of the ALT Pathway

**Figure 5:**
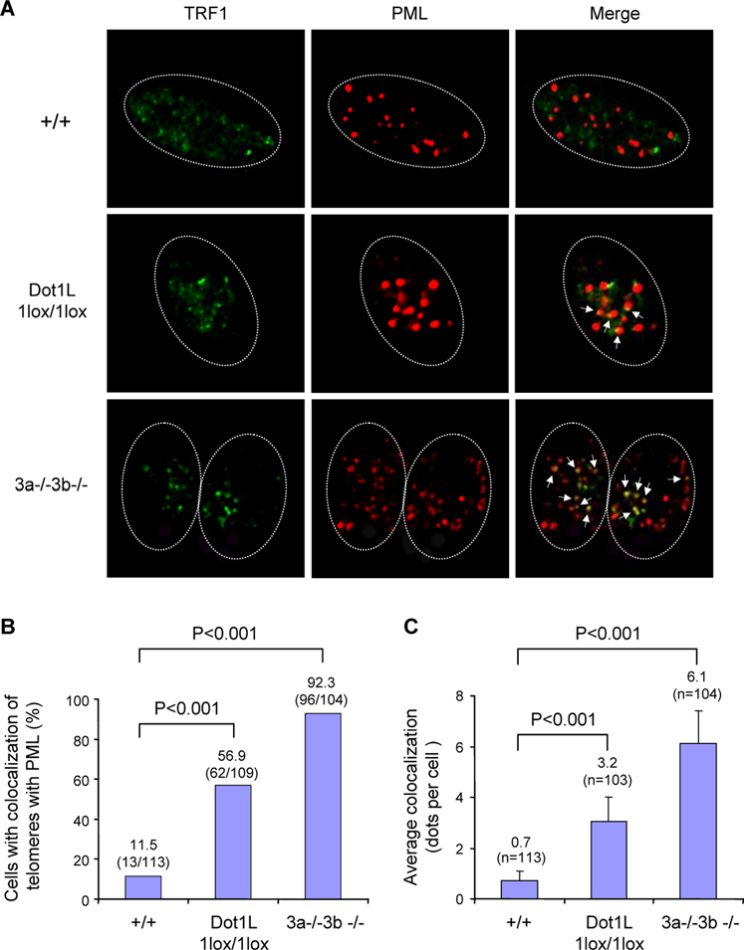
Increased APBs in *Dot1L*-deficient cells. (A) Confocal microscopy images showing either TRF1 (telomere marker, green), PML (marker for PML bodies, red), or combined fluorescence (yellow if colocalize, indicated by arrows) in wild-type (+/+) and *Dot1L*-deficient (1lox/1lox) ES cells. Late-passage (p120) *Dnmt3a/3b*-deficient (3a−/−3b−/−) ES cells were used as a positive control. Circled are nuclei of cells. (B) Quantification of percentage of cells showing colocalization of telomeres with PML bodies. A cell was considered positive when it showed 2 or more colocalization events. An increased frequency of cells showing APBs was observed in *Dot1^1lox/1lox^* cultures compared to wild-type controls (χ^2^ test, P<0.001). (C) Quantification of the number of APBs per cell. *Dot1^1lox/1lox^* cells showed a significant increase in the number of APBs compared to wild-type cells (χ^2^ test, P<0.001).

Our data suggested a role for Dot1L in the homeostasis of telomere length. Two main mechanisms have been described for the maintenance of mammalian telomeres: the addition of telomeric repeats by telomerase and the so-called alternative lengthening of telomere (ALT) mechanism that relies on homologous recombination between telomeric sequences [15],[16]. *Dot1L* mutant ES cells showed increased telomere heterogeneity (Figure 4), which is a hallmark of ALT cells [17]. To determine whether the ALT pathway is activated in *Dot1L*-deficient cells, we assessed the presence of ALT-associated PML bodies (APBs, colocalization of PML and telomeres), another hallmark of ALT [17]. *Dot1L^+/+^*, *Dot1L^1lox/1lox^*, as well as Dnmt3a−/−3b−/− (positive control) ES cells were immunostained with antibodies against TRF1 (a telomere-binding protein) and PML. In the absence of Dot1L, both the frequency of cells showing APBs and the number of APBs per cell were significantly increased compared to wild-type cells (Figure 5, A–C, χ^2^ tests, P<0.001), suggesting that aberrant elongation of telomeres in *Dot1L*-deficient cells was due, at least in part, to activation of the ALT pathway.

### Dot1L Deficiency Results in Loss of Heterochromatin Marks at Telomeres and Centromeres

**Figure 6:**
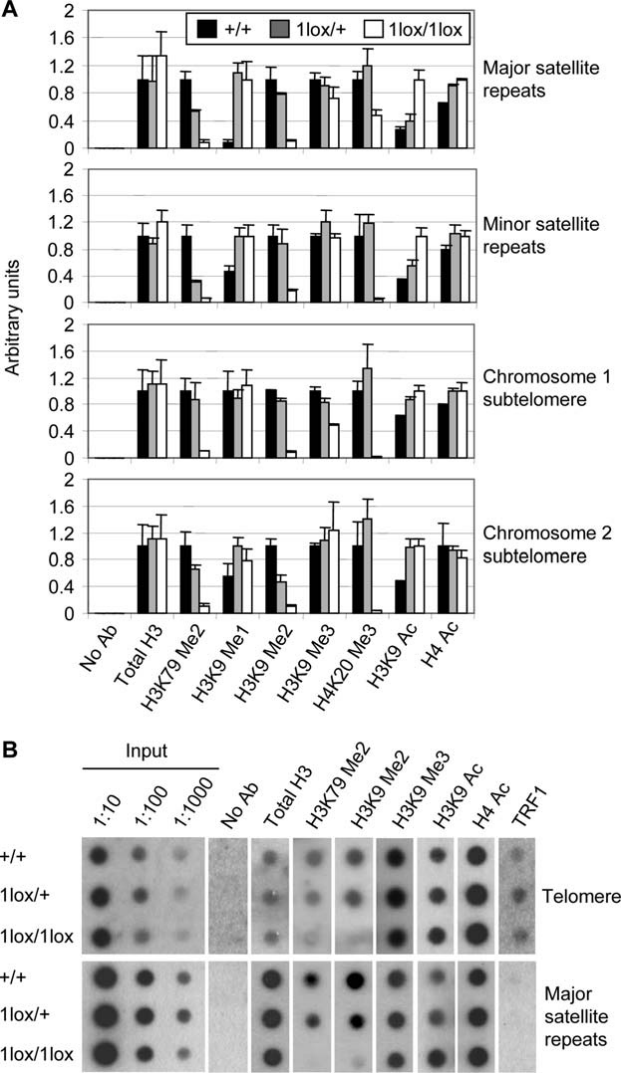
Changes of heterochromatin structure in *Dot1L*-deficient ES cells. (A) Quantitative real-time PCR results using DNA from *Dot1L* mutant and wild-type ES cells immunoprecipitated with antibodies specific for the indicated histone modifications or without an antibody (No Ab) and normalized using input DNA values. PCR primers specific for major satellite repeats, minor satellite repeats, the subtelomere region of chromosome 1 or the subtelomere region of chromosome 2 were used. (B) Dot blot analysis of ChIP DNA using either a telomere-specific probe or a major satellite repeat-specific probe. Input DNA at 1∶10, 1∶100 and 1∶1000 dilutions was used as a positive control. DNA precipitated from 2.5×10^6^ cells were used for each assay.

Aneuploidy and telomere elongation can result from defects in the chromatin structure at centromeres and telomeres, respectively [18]–[23]. To evaluate changes in chromatin structure in *Dot1L* mutant cells, we used chromatin immunoprecipitation (ChIP) to examine histone modifications at major satellite repeats (present at pericentric regions), minor satellite repeats (present at centromeric regions), telomeric repeats, and subtelomeric regions (Figure 6). H3K79 di-methylation was detected in all these heterochromatin regions in *Dot1L^+/+^* cells (Figure 6, A and B). As expected, this modification was reduced in *Dot1L^1lox/+^* cells and almost absent in *Dot1L^1lox/1lox^* cells (Figure 6, A and B), validating our experimental procedures. As further controls, the levels of centromere- and telomere-bound histone H3 were similar in wild-type and *Dot1L* mutant cells (Figure 6, A and B), and the telomere-binding protein TRF1 associated with telomeric repeats, but not with major satellite repeats (Figure 6B).

In *Dot1L^1lox/1lox^* cells, H4K20 tri-methylation, a hallmark of constitutive heterochromatin, was greatly reduced at minor satellite repeats and sub-telomere regions, and moderately reduced at major satellite repeats (Figure 6A). Consistent with this observation, immunofluorescence analysis revealed the loss of enrichment of H4K20 tri-methylation at pericentric heterochromatin in the absence of Dot1L (Figure S3). H3K9 di-methylation, but not H3K9 tri-methylation, was reduced in all regions examined in *Dot1L^1lox/1lox^* cells (Figure 6, A and B). Concomitantly, H3K9 mono-methylation showed marked increases at major satellite repeats and minor satellite repeats (Figure 6A), and H3K9 acetylation, a mark of euchromatin, was elevated in all regions examined (Figure 6, A and B). These changes appeared to be heterochromatin-specific, as all histone modifications examined, except H3K79 methylation, showed no global changes in *Dot1L* mutant cells (Figure S4). Dot1L deficiency did not cause alterations in DNA methylation at major satellite repeats and minor satellite repeats as well as other genomic regions such as the intracisternal A-particle (IAP) retroviral elements (Figure S5). Altogether, these results suggest that loss of H3K79 methylation results in a less compacted (or more open) chromatin state at centromeres and telomeres.

## Discussion

In this report, we provide genetic evidence that Dot1L and, by implication, H3K79 methylation are essential for mammalian development and normal cellular function. We show that loss of Dot1L results in yolk sac angiogenesis defects and embryonic lethality. Furthermore, our characterization of *Dot1L*-deficient ES cells reveals that, like in yeast, H3K79 methylation plays a critical role in heterochromatin structure in mammalian cells. Considering the differences in chromatin structure between yeast and mammals, the phenotypic similarities in mutants of these organisms are both striking and surprising. For example, both mutant organisms exhibit telomere elongation, but mammalian telomeres contain H3K79 methylated histones, while *S. cerevisiae* telomeres contain no histones at all. Furthermore, while ∼7% of the budding yeast genome is packaged as heterochromatin (including rDNA), ∼55% of the mammalian genome is composed of heterochromatin [24].

Dot1L recruitment is coupled with gene transcription [25] and H3K79 methylation is enriched in euchromatin, which seem to be counterintuitive to the heterochromatin phenotype of *Dot1L*-deficient cells. One possible explanation is that *Dot1L* inactivation alters the expression of specific factors involved in heterochromatin assembly. Alternatively, global loss of H3K79 methylation may result in redistribution of heterochromatin factors, thereby reducing their relative abundance at constitutive heterochromatin. Indeed, loss of Dot1 in yeast leads to mislocalization of the Sir proteins, which promote heterochromatin formation and telomere silencing [7],[8].

H3K9 tri-methylation and H4K20 tri-methylation are hallmarks of constitutive heterochromatin, such as that at centromeres and telomeres [20],[21],[23],[26],[27]. Based on the observation that H3K9 tri-methylation by the Suv39h methyltransferases is required for the induction of H4K20 tri-methylation by the Suv4-20h methyltransferases at pericentric heterochromatin, a sequential model of chromatin assembly at constitutive heterochromatin has been proposed in which Suv4-20h enzymes act downstream of the Suv39h enzymes [26]. *Dot1L*-deficient cells show loss of H4K20 tri-methylation at telomeres and centromeres, suggesting that Dot1L functions upstream of the Suv20h enzymes. Given that H3K9 tri-methylation shows no obvious alterations in *Dot1L*-deficient cells, it is possible that Dot1L acts in parallel or downstream of the Suv39h enzymes. Interestingly, despite the relatively normal levels of H3K9 tri-methylation, H3K9 di-methylation is severely reduced at constitutive heterochromatin in the absence of Dot1L. Because the total levels of H3K9 di-methylation and of several H3K9 methyltransferases (Suv39h1, ESET, and G9a) are not altered in *Dot1L*-deficient cells (Figure S4), we speculate that Dot1L deficiency may affect the targeting of one or more H3K9 methyltransferases or demethylases to constitutive heterochromatin. Further studies will be required to elucidate the mechanisms by which Dot1L and H3K79 methylation regulate heterochromatin.

Perturbation of epigenetic marks at constitutive heterochromatin has been shown to cause chromosome instability and telomere elongation [20]–[23]. Therefore, aberrant changes in chromatin at centromeres and telomeres most likely underlie the aneuploidy and telomere elongation observed in *Dot1L*-deficient ES cells. How the observed alterations in chromatin structure and cellular function contribute to the developmental abnormalities in *Dot1L*-deficient embryos is less clear. The requirement of Dot1L for normal cellular function does not appear to be ES cell-specific, as RNAi-mediated Dot1L knock-down in somatic cell lines also leads to growth arrest and cell death [13]. It is thus probable that intrinsic defects in cellular proliferation and viability, which themselves are likely the result of heterochromatin alterations, contribute to the growth defects and apoptosis observed in *Dot1L* mutant embryos. However, we believe that yolk sac defects are a major cause of embryonic lethality. In the absence of Dot1L, yolk sac angiogenesis is severely impaired. As both the endoderm and mesoderm cell layers of the visceral yolk sac are critical for blood vessel development [28],[29] and both express Dot1L, we speculate that, in the absence of Dot1L, aberrant changes in gene expression and chromatin structure in one or both cell layers may underlie the yolk sac vascular defects. Some embryonic abnormalities, such as cardiac dilation, could be secondary to yolk sac vascular defects. Although Dot1L is also expressed in primitive erythrocytes, loss of Dot1L does not appear to have an obvious impact on erythropoiesis. It remains to be determined, however, whether the erythrocyte function is impaired.

## Materials and Methods

### Construction of the Gene Targeting Vector

The *Dot1L* conditional targeting vector, in which a 2.3-kb genomic region containing exons 5 and 6 was flanked by *loxP* sites, was constructed by sequentially subcloning *Dot1L* genomic fragments and a floxed βGeo cassette into pBluescript SK (Stratagene). The *Dot1L* genomic fragments were generated by PCR using mouse genomic DNA as the template. The primer pairs used were: 5′-TTC ACT AGT CCC CAC CTT TGG ATT G-3′ and 5′-GGC ACT AGT GTC ACA CAC CTT TA-3′ for the 5′ arm, 5′-CAT GTC GAC ACC GTG TAG TCC TGG TGG GA-3′ and 5′-CTC GGC CGG CCT TGC CTG TGG CTG ACG-3′ for the 3′ arm, and 5′-GAC ACC GGT GCC TGG CAA CCT TTT GG-3′ and 5′-CTG GGC GCG CCA CCA GGA ACA CAC AGG TAC-3′ for the floxed region (underlined are the restriction sites used for cloning). The identity of the vector was verified by DNA sequencing.

### Generation of *Dot1L* Mutant Mice

The *Dot1L* conditional targeting vector was transfected into ES cells via electroporation, and transfected cells were selected with G418. Clones with homologous recombination (*Dot1L^3lox/+^*) were identified using Southern blot. Genomic DNA was digested with *Eco*RI and hybridized with a 5′ external probe (The probe was generated by PCR using the following primers: 5′-CTC TGG TAC CTT TGT TGT TAT ACA G-3′ and 5′-CTC TCA AGT CGA CTG TAA GAT GAA G-3′). Multiple *Dot1L^3lox/+^* clones were used to generate chimeric mice and F1 heterozygotes. Deletion of exons 5 and 6 as well as the βGeo cassette was achieved by crossing *Dot1L^3lox/+^* mice with Zp3-Cre transgenic mice, which express the Cre recombinase in the germline. Mutant mice were maintained on a C57BL/6 inbred or a C57BL/6-129Sv hybrid background. Primers used for PCR genotyping were: DF1: 5′-GGA ACT CAA GCT ATA GAC AG-3′, DR1: 5′-CAC TGC CCA GGT CGA CAA ACA G-3′, and DR2: 5′-ATC CTC TCT CCT GAG GAG GCA GC-3′ (Figure 1).

### Embryo Collection, X-Gal Staining, and Histology

Female mice in *Dot1L^1lox/+^* intercrosses were examined for plug formation to establish the timing of copulation. Deciduas were isolated from euthanized females at various time points following copulation, and embryos were examined under a dissecting microscope. DNA from the yolk sac was used for genotyping by PCR using the primers described above. X-gal staining was performed on 7.5- to 12.5-dpc *Dot1L^3lox/+^* embryos and littermates as previously described [30]. Embryo, yolk sac, and placental tissue specimens, which were harvested at 9.5-dpc, were fixed in Bouin's solution, washed extensively in 70% ethanol, processed routinely for paraffin embedding, sectioned at 5 µm, stained with hematoxylin and eosin, and then evaluated by bright field microscopy.

### ES Cell Derivation and Culture

*Dot1L* mutant ES cells were derived from blastocysts produced from intercrosses of *Dot1L^1lox/+^* mice, as previously described [31]. Established ES lines were maintained in ES cell medium [32]. Apoptosis was analyzed using an Annexin V-PE apoptosis detection kit (BD Pharmingen). Cell cycle analysis was done using a PI/RNase Staining Buffer (BD Pharmingen).

### Immunofluorescence and Immunoblot Analyses

Immunoblot and indirect immunofluorescence analyses were carried out using standard procedures. The following antibodies were used: anti-H3K79Me1 (Abcam), anti-H3K79Me2 (Abcam), anti-H3K79Me3 (Abcam), anti-H3 (Millipore), anti-H3K4Me2 (Millipore), anti-H3K4Me3 (Millipore), anti-H3K9Me1 (Millipore), anti-H3K9Me2 (Millipore), anti-H3K9Me3 (Millipore), anti-H3K27Me1 (Millipore), anti-H3K27Me3 (Millipore), anti-H3K9Ac (Millipore), anti-H4K20Me3 (Millipore), anti-H4Ac (Millipore), anti-Suv39h1 (Upstate), anti-ESET (Upstate), anti-G9a (Cell Signaling), anti-TRF1 (Abcam), anti-PML (Chemicon), Alexa 488-conjugated goat anti-rabbit IgG (Molecular probes), Alexa 555-conjugated goat anti-mouse IgG (Molecular Probes), and peroxidase-conjugated goat anti-rabbit and goat anti-mouse IgG (Jackson ImmunoResearch Laboratories).

### Mass Spectrometry Analysis

Histone H3 purified from ES cells was digested with trypsin, and the resulting peptides were analyzed using a LTQ-FT mass spectrometer (Thermo Fisher Scientific Inc.) hyphenated with an Agilent 1200 HPLC system (Agilent). Identification of the peptides was performed by searching the MS/MS fragmentation data against the histone H3 sequence using MASCOT search software (Matrix Science, version 2.1). All identifications were manually inspected for correctness. The abundance of each identified and validated peptide was calculated from its peak intensity using extracted ion chromatogram (XIC) of LC/MS spectra. Relative quantification of different forms of H3K79 methylation was performed by comparing the signal intensities of the tryptic peptide EIAQDFKTDLR at *m/z* 668.35 ([MH_2_]^2+^), EIAQDFKmeTDLR at *m/z* 675.36 ([MH_2_]^2+^), EIAQDFK2meTDLR at *m/z* 682.35 ([MH_2_]^2+^), and EIAQDFK3meTDLR at *m/z* 689.35 ([MH_2_]^2+^).

### Telomere Length Analysis

To analyze telomere length, we performed Q-FISH and TRF analyses according to procedures described previously [22].

### Metaphase Spread Analysis

To prepare metaphase spreads, cells were incubated with 0.1 µg/ml of colcemid for 4 hours and then harvested and resuspended in 200 µl PBS. 10 ml of 75 mM KCl solution was added dropwise with constant gentle agitation. Cells were fixed by slow addition of 3∶1 methanol/acetic acid solution, and then dropped onto a microscope slide. Slides were washed in 70% acetic acid, stained with DAPI and mounted. Chromosome spreads were observed using a Zeiss fluorescence microscope.

### Chromatin Immunoprecipitation Assays

ChIP was performed using 20×10^6^ ES cells as described in the online protocol provided by Upstate. Antibody sources are described above. Purified DNA was either analyzed with quantitative real-time PCR (qPCR) using Applied Biosystems SYBR PCR mastermix or used in a dot blot assay as described [22]. qPCR primers used were specific for major satellite repeats, minor satellite repeats [33], and subtelomeric regions of chromosome 1 (forward: 5′-TTA GGA CTT CTG GCT TCG GTA G-3′, reverse: 5′-AGC TGT GGC AGG CAT CGT GGC-3′) and chromosome 2 (forward: 5′-GAA TCC TCC CTG TAG CAG GG-3′, reverse: 5′-GTA CAT AAC CGA TCC AGG TGT G-3′). Relative enrichment was calculated as 2ˆ (C_T_ (control CHIP) - C_T_ (experimental CHIP)), where C_T_ is equal to the C_T_ (immunoprecipitated sample) - C_T_ (input) and normalized so that the wild-type value was 1, with the exception of H3K9Me1 at major satellite repeats where the *Dot1L^1lox/1lox^* value was 1. Each sample used in the dot blot contained DNA precipitated from 2.5×10^6^ cells. Probes used were ^32^P-labelled oligonucleotides specific for telomeric repeats ((TTAGGG)_x11_) and major satellite repeats (5′-TAT GGC GAG GAA AAC TGA AAA AGG TGG AAA ATT TAG AAA TGT CCA CTG TAG GAC GTG GAA TAT GGC AAG-3′), respectively.

### DNA Methylation Assay

Genomic DNA isolated from ES cells was digested with methylation-sensitive restriction enzymes and analyzed by Southern hybridization using probes specific for the major satellite repeats, the minor satellite repeats, and the intracisternal A particle retrovirus [32],[34].

## Supporting Information

Figure S1 Elevated apoptosis in *Dot1L^1lox/1lox^* embryos. Representative hematoxylin and eosin-stained sections from 9.5-dpc *Dot1L^+/+^* (left) and *Dot1L^1lox/1lox^* (right) embryos illustrating focal areas of extensive apoptosis in the *Dot1L^1lox/1lox^* embryo (asterisk). Scale bars = 100 µm.
(0.5 MB PDF)

Figure S2 Identification of H3K79 methylation by Nano-ESI MS/MS. Tryptic digest mixtures were analyzed by ESI MS using an LTQ-FT instrument. The precursor ion (m/z = 668.35), corresponding to the doubly charged (z = 2) version of peptide ion was selected for collision-induced dissociation (CID)-based MS/MS analysis. The fragment ion spectrum was inspected for y ions and the deduced sequence is indicated. The double methylation on K79 was identified from the spectrum. Unmodified, mono-methylated, and tri-methylated peptides were identified in the same way (data not shown).
(0.5 MB PDF)

Figure S3 Loss of H4K20Me3 enrichment at pericentric heterochromatin in *Dot1L^1lox/1lox^* cells. *Dot1L^+/+^*, *Dot1L^1lox/+^* and *Dot1L^1lox/1lox^* ES cells were immunostained with antibodies specific for the indicated histone modifications and examined using a fluorescent microscope. *Dot1L^1lox/1lox^* cells showed no obvious alterations in the level and localization pattern of all modifications tested, with the exception of H4K20 tri-methylation, which displayed a more diffused nuclear pattern compared to *Dot1L^+/+^* and *Dot1L^1lox/+^* cells.
(0.2 MB PDF)

Figure S4 No global changes in histone modifications besides H3K79 methylation in *Dot1L^1lox/1lox^* cells. Lysates from *Dot1L^+/+^*, *Dot1L^1lox/+^* and *Dot1L^1lox/1lox^* ES cells were analyzed with immunoblotting using antibodies specific for the indicated histone modifications (A) or H3K9 methyltransferases (B).
(0.1 MB PDF)

Figure S5 No alteration in DNA methylation in the absence of Dot1L. Genomic DNA from *Dot1L^+/+^*, *Dot1L^1lox/+^*, *Dot1L^1lox/1lox^*, and *Dnmt1−/−* (c/c) ES cells were digested with *Mae*II (for major satellite repeats) or *Hpa*II (for minor satellite repeats and IAP) and analyzed by Southern blot using the indicated probes.
(0.02 MB PDF)
