# Supplementary figures and images for: The Histone H3K79 Methyltransferase Dot1L Is Essential for Mammalian Development and Heterochromatin Structure
Source: PLoS Genet. 2008 Sep 12;4(9):e1000190. doi: 10.1371/journal.pgen.1000190 (PMC2527135; doi:10.1371/journal.pgen.1000190)

**+/+**

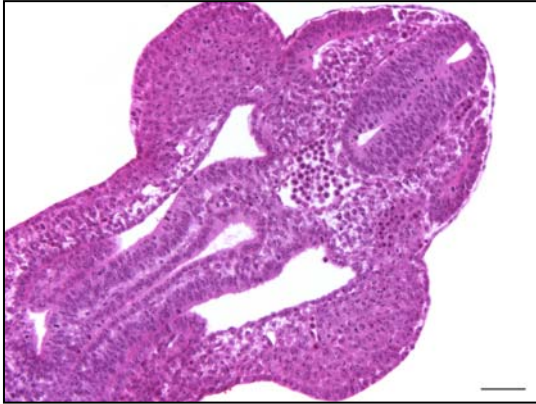

**1lox/1lox**

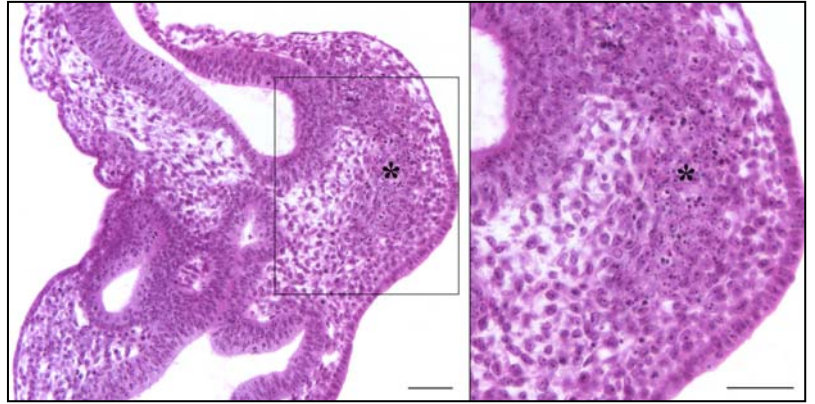

Supplement: Figure S1 — Elevated apoptosis in Dot1L1lox/1lox embryos. Representative hematoxylin and eosin-stained sections from 9.5-dpc Dot1L+/+ (left) and Dot1L1lox/1lox (right) embryos illustrating focal areas of extensive apoptosis in the Dot1L1lox/1lox embryo (asterisk). Scale bars = 100 µm. (0.5 MB PDF) [file pgen.1000190.s001.pdf]

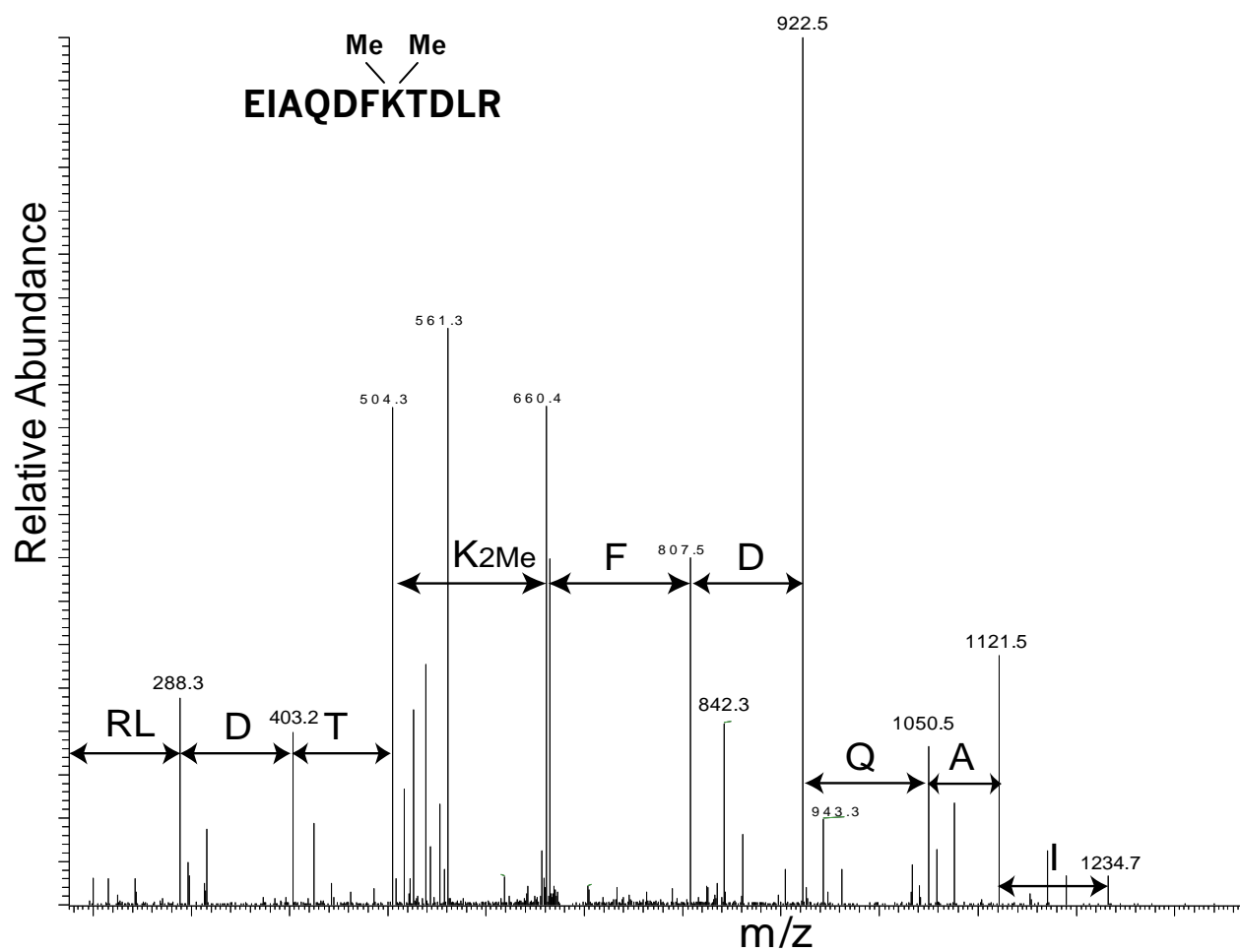

Supplement: Figure S2 — Identification of H3K79 methylation by Nano-ESI MS/MS. Tryptic digest mixtures were analyzed by ESI MS using an LTQ-FT instrument. The precursor ion (m/z = 668.35), corresponding to the doubly charged (z = 2) version of peptide ion was selected for collision-induced dissociation (CID)-based MS/MS analysis. The fragment ion spectrum was inspected for y ions and the deduced sequence is indicated. The double methylation on K79 was identified from the spectrum. Unmodified, mono-methylated, and tri-methylated peptides were identified in the same way (data not shown). (0.5 MB PDF) [file pgen.1000190.s002.pdf]

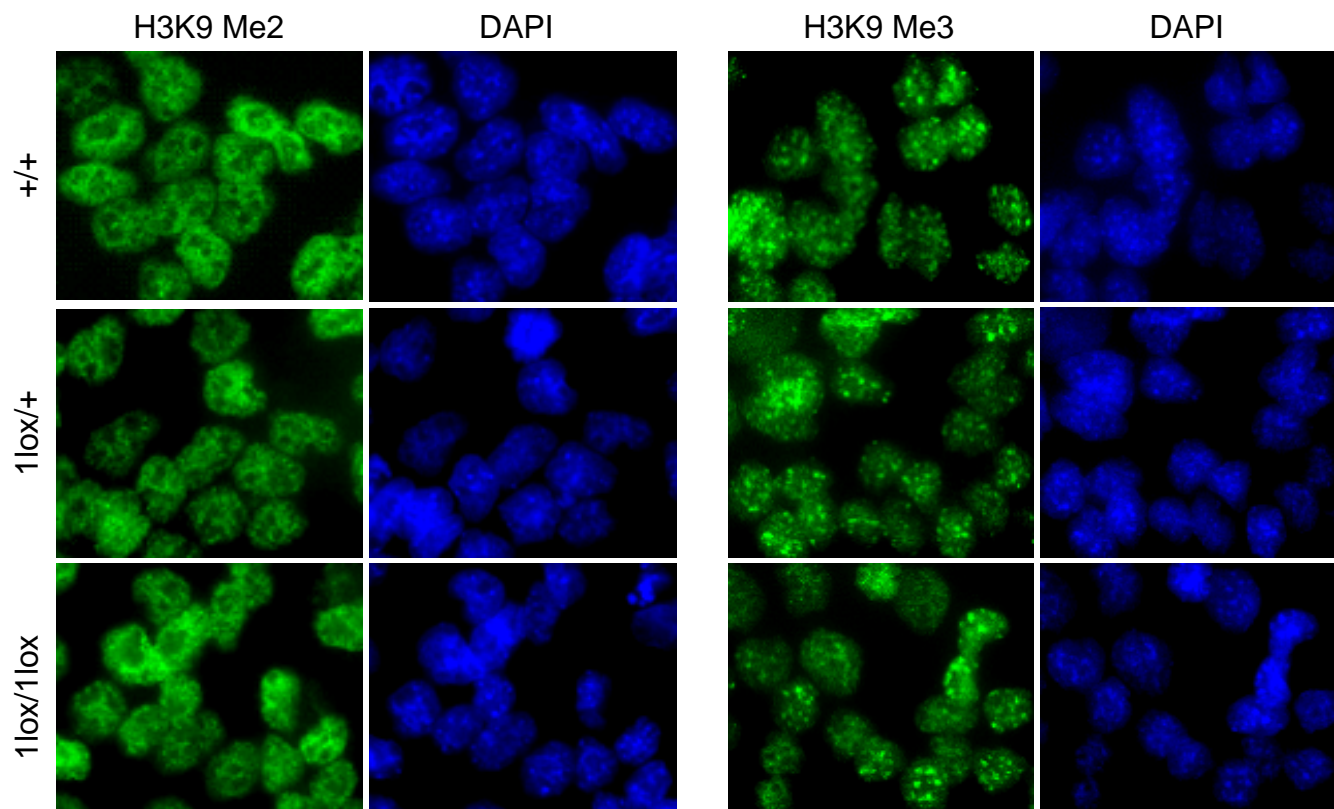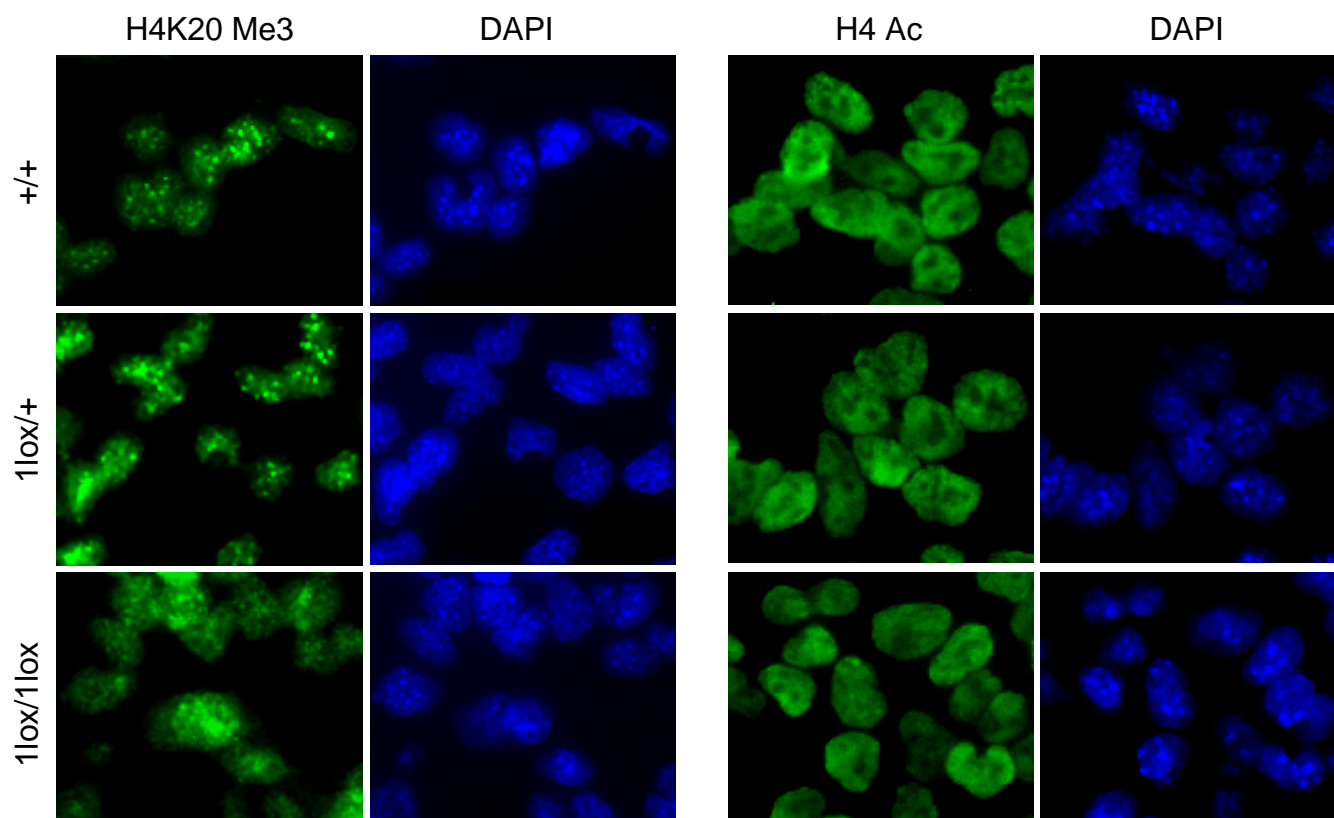

Supplement: Figure S3 — Loss of H4K20Me3 enrichment at pericentric heterochromatin in Dot1L1lox/1lox cells. Dot1L+/+, Dot1L1lox/+ and Dot1L1lox/1lox ES cells were immunostained with antibodies specific for the indicated histone modifications and examined using a fluorescent microscope. Dot1L1lox/1lox cells showed no obvious alterations in the level and localization pattern of all modifications tested, with the exception of H4K20 tri-methylation, which displayed a more diffused nuclear pattern compared to Dot1L+/+ and Dot1L1lox/+ cells. (0.2 MB PDF) [file pgen.1000190.s003.pdf]

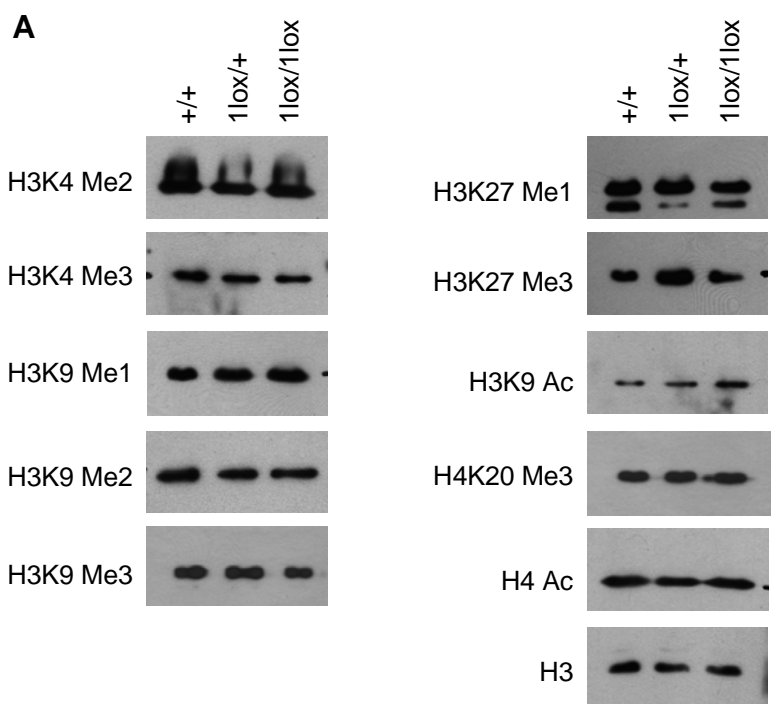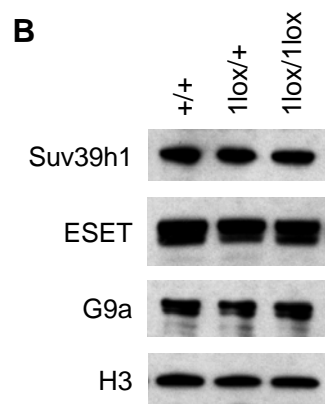

Supplement: Figure S4 — No global changes in histone modifications besides H3K79 methylation in Dot1L1lox/1lox cells. Lysates from Dot1L+/+, Dot1L1lox/+ and Dot1L1lox/1lox ES cells were analyzed with immunoblotting using antibodies specific for the indicated histone modifications (A) or H3K9 methyltransferases (B). (0.1 MB PDF) [file pgen.1000190.s004.pdf]

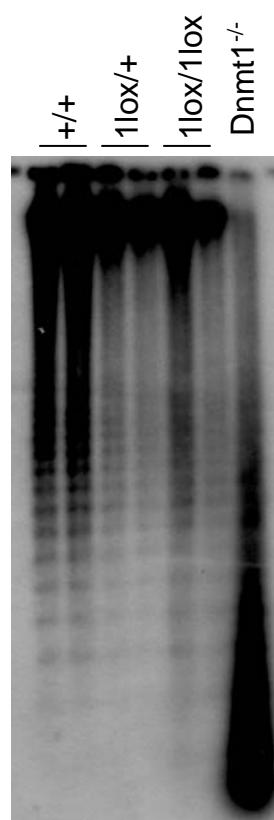

Major sat. repeat

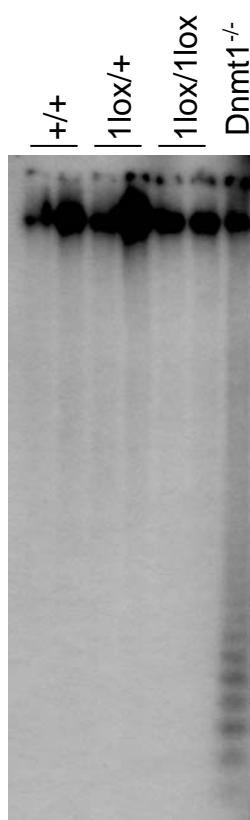

Minor sat. repeat

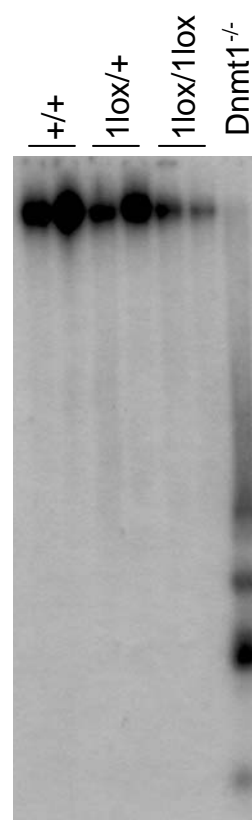

IAP

Supplement: Figure S5 — No alteration in DNA methylation in the absence of Dot1L. Genomic DNA from Dot1L+/+, Dot1L1lox/+, Dot1L1lox/1lox, and Dnmt1−/− (c/c) ES cells were digested with MaeII (for major satellite repeats) or HpaII (for minor satellite repeats and IAP) and analyzed by Southern blot using the indicated probes. (0.02 MB PDF) [file pgen.1000190.s005.pdf]
